# Determination of the complete mitochondrial genome of *Pseudobagrus gracilis* (Siluriformes: Bagridae) and its phylogeny

**DOI:** 10.1080/23802359.2021.1951626

**Published:** 2021-07-15

**Authors:** Shuli Zhu, Jie Li

**Affiliations:** a Pearl River Fisheries Research Institute, Chinese Academy of Fishery Sciences, Guangzhou, China; b Key Laboratory of Aquatic Animal Immune Technology of Guangdong Province, Guangzhou, China; c Guangzhou Scientific Observing and Experimental Station of National Fisheries Resources and Environment, Guangzhou, China

**Keywords:** *Pseudobagrus gracilis*, mitochondrial genome, phylogenetic analyses

## Abstract

*Pseudobagrus gracilis* is an endemic bagrid catfish in the Pearl River. To date, sparse studies conducted on this species have blocked our understanding of this species. In this study, the complete mitochondrial genome of *P. gracilis* was sequenced and reported using Illumina MiSeq platform. The *P. gracilis* mitogenome was 16,527 bp in length and comprised 13 protein-coding genes, 22 transfer RNA genes, 2 ribosomal RNA genes, and one control region (D-loop). Its overall nucleotide base composition was 31.0% (A), 15.6% (G), 26.3% (C), and 27.1% (T), with an AT content 58.1%. Phylogenetic analyses based on Neighbor-joining approach revealed that *Pseudobagrus* species formed three lineages (I, II and III) and *P. gracilis* had close relationship with *P. emarginatus*, *P. pratti* and *P. truncatus*.

## Introduction

*Pseudobagrus gracilis* (Li et al. 2005), a bagrid catfish that only distributed in the Pearl River, was firstly detected and described in 2005 (Li et al. 2005). This species has been confused or misidentified with two other *Pseudobagrus* species, e.g., *P. pratti* and *P. adiposalis*, which also inhabit in the drainages in the Southern China (Pan 1984; Zhu 1995). Currently, with exception of the first description in 2005 (Li et al. 2005), no study has been conducted for *P. gracilis*. We determined the whole mitochondrial genome of *P. gracilis* using next generation sequencing technology and tried to reveal its phylogenetic relationships among *Pseudobagrus* species.

A sample of *P. gracilis* was collected in June 2020 from the farm market in Pingle County (24.627 N, 110.649E), Guilin City, Guangxi Province, China. We clipped a fraction of fin tissue for genomic DNA extraction and extracted total genomic DNA from fin tissues using a Genomic DNA Isolation Kit (QiaGene, Germany). A specimen and genomic DNA sample were deposited at the fish collection of Pearl River Fisheries Research Institute, Chinese Academy of Fishery Sciences (https://www.prfri.ac.cn/, Jie Li, lijie1561@163.com) under the voucher number XSNC2020001. The Illumina MiSeq platform (Illumina Inc, San Diego, CA, USA) was employed to sequence the complete mitochondrial genome and the software SPAdes 3.9.0 was used to assemble the raw sequence reads into contigs (Bankevich et al. 2012). The complete mitochondrial genome was achieved using the contigs in the software SOAPdenovo (Luo et al. 2012) and annotated the protein-coding genes and rRNA genes using web server DOGMA (Wyman et al. 2004).

The length of the *P. gracilis* mitogenome (GenBank nos: MW980438) was 16,527 base pairs (bp). Its overall nucleotide base composition was 31.0% (A), 15.6% (G), 26.3% (C), and 27.1% (T) and thus the overall AT content was 58.1%, which was basically consistent with the results of other published *Pseudobagrus* genomes (Pan et al. 2015; Wei et al. 2015; Yang et al. 2016; Zou et al. 2019). The mitogenome included 13 protein-coding genes, 2 rRNA genes, 22 tRNA genes and a control region (D-loop). Gene overlaps were detected at seven gene junctions from 1 to 11 bp and involved a total of 37 bp. Among these mitochondrial genes, eight *tRNA* genes (*tRNA-Gln, tRNA-Ala, tRNA-Asn, tRNA-Cys, tRNA-Tyr, tRNA-Ser, tRNA-Glu* and *tRNA-Pro*) and a protein coding gene (*ND6*) were encoded on the L-strand, while other genes were encoded on the H-strand. All protein-coding genes began with the typical start codon ATG, with the exception of *COI*, which started with GTG. Six protein-coding genes ended with a complete TAA (*COI*, *ATP8*, *ATP6*, *ND4L*, *ND5* and *ND6*) and two genes finished with a complete TAG (*ND1* and *ND2*). Furthermore, five genes (*COII*, *COIII*, *ND3*, *ND4* and *CYTB*) had incomplete stop codon T.

**Figure 1.**
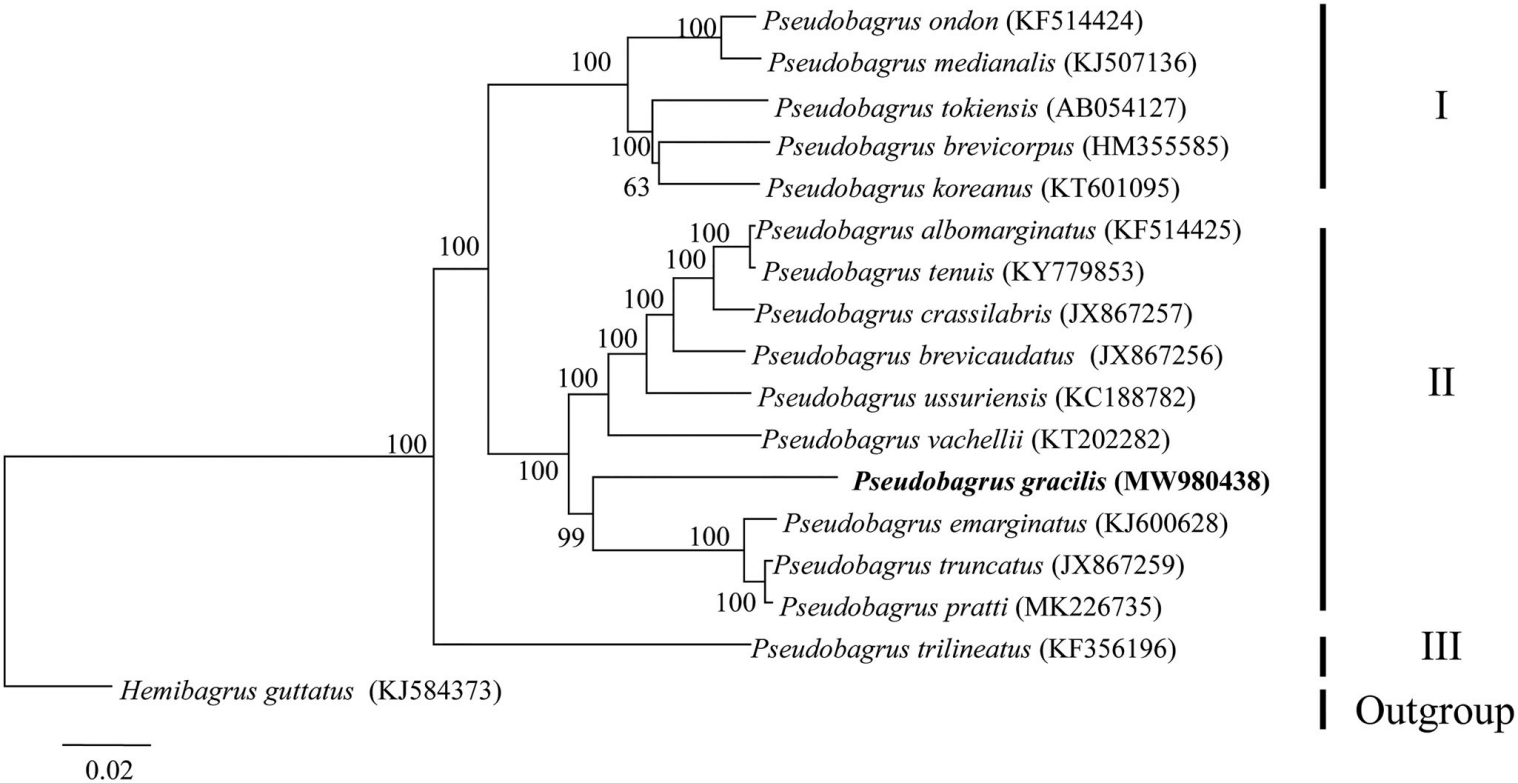
Phylogenetic trees based neighbor-joining showing the phylogenetic relationships among *Pseudobagrus* mitogenomes based on 13 protein-coding genes. Values on branches indicate bootstrap values from neighbor-joining tree.

Phylogenetic tree of genus *Pseudobagrus* was constructed based on 13 protein coding genes of 16 *Pseudobagrus* mitogenomes and one outgroup (*Hemibagrus guttatus*) using Neighbor-joining method in MEGA 6 (Tamura et al. 2013). Neighbor-joining tree yielded three well supported major lineages (Lineage I, II and III; Figure 1), which gain one more lineage than the previous studies (Yang et al. 2016; Zou et al. 2019). The most discordant result compared with previous researches in our study was that *P. trilineatus* generated an independent lineage and located at the base of the other two lineages (Figure 1). The studied species *P. gracilis* had close relationship with *P. emarginatus*, *P*. *pratti* and *P. truncatus* (Figure 1).

## Ethical approval

Experiments were performed in accordance with the recommendations of the Ethics Committee of Pearl River Fisheries Research Institute, Chinese Academy of Fishery Sciences. These policies were enacted according to the Chinese Association for the Laboratory Animal Sciences and the Institutional Animal Care and Use Committee (IACUC) protocols.

## Data Availability

The genome sequence data that support the findings of this study are openly available in GenBank of NCBI at (https://www.ncbi.nlm.nih.gov/) under the accession no. MW980438. The associated BioProject, SRA, and Bio-Sample numbers are PRJNA723410, SRR14291144 and SAMN18816459, respectively.
